# Application of DPSIR model to identify the drivers and impacts of land use and land cover changes and climate change on land, water, and livelihoods in the L. Kyoga basin: implications for sustainable management

**DOI:** 10.1186/s40068-022-00254-8

**Published:** 2022-05-19

**Authors:** John Peter Obubu, Robinson Odong, Tena Alamerew, Tadesse Fetahi, Seyoum Mengistou

**Affiliations:** 1 African Centre of Excellence for Water Management, Addis Ababa University, P. O. Box 1176, Addis Ababa, Ethiopia; 2 Department of Water Quality Management, Directorate of Water Resources Management, Ministry of Water and Environment, P. O. Box 20026, Kampala, Uganda; 3 Department of Zoology, Entomology and Fisheries Sciences, College of Natural Sciences, Makerere University, Kampala, Uganda; 4 Ethiopian Institute of Water Resource, Addis Ababa University, Addis Ababa, Ethiopia

**Keywords:** Drivers, Communities, Forests, Wetlands, Fertilizers, Impacts, Population

## Abstract

Land use, land cover, and climate change impacts are current global challenges that are affecting many sectors, like agricultural production, socio-economic development, water quality, and causing land fragmentation. In developing countries like Uganda, rural areas with high populations dependent on agriculture are the most affected. The development of sustainable management measures requires proper identification of drivers and impacts on the environment and livelihoods of the affected communities. This study applied drivers, pressure, state, impact, and response model in the L. Kyoga basin to determine the drivers and impacts of land use, land cover, and climate change on livelihoods and the environment. The objective of this study was to determine the drivers and impacts of land use, land cover, and climate changes on the environment and livelihoods in the L. Kyoga basin and suggest sustainable mitigation measures. Focus group discussions, key informant interviews, field observations, and literature reviews were used to collect data. Population increase and climate change were the leading drivers, while agriculture and urbanization were the primary pressures, leading to degraded land, wetlands, and forests; loss of soil fertility, hunger, poverty, poor water quality, which are getting worse. The local communities, government, and non-government institutions had responses to impacts, including agrochemicals, restoration, and conservation approaches. Although most responses were at a small/pilot scale level, most responses had promising results. The application of policies and regulations to manage impacts was also found to be weak. Land use, land cover changes, and climate change occur in the L. Kyoga basin with major impacts on land, water, and community livelihoods. With the observed increase in climate change and population growth, drivers and impacts are potentially getting worse. Therefore, it is essential to expand interventions, provide relief, review policies and regulations, and enforce them. The findings are helpful for decisions and policy-makers to design appropriate management options.

## Introduction

Land use and land cover (LULC) change and climate change (CC) have become synonymous with contemporary discussions globally as they are intertwined and are impacting many aspects of livelihoods and socio-economic developments (IPCC 2007; Wan et al. 2014; Amjath-Babu et al. 2016). Whereas land cover (LC) includes the natural physical features of the land in addition to artificial structures that form the landscape, land use (LU) covers the way humans utilize land and its associated resources (Alemayehu et al. 2009). On the other hand, climate change refers to the human-induced change in climate that varies the configuration of the global atmosphere and adds to natural variability experienced over similar time scales (UNFCCC 2011). There are many drivers of LULC and CC at the international level, and they are divided into two main categories, i.e., proximate and underlying (Bisaro et al. 2014; Pingali et al. 2014; Belay et al. 2015). The proximate drivers with direct impacts on watersheds include natural phenomena associated with climate, droughts, topography, deforestation, agriculture, and wildfires (Dregne 2002; Lambin et al. 2008). The underlying drivers, with indirect consequences, include population density, poverty, the land tenure system, and weakly implemented regulations and policies (Tiffen et al. 1994; Kabubo-Mariara 2007; Gessessew 2017). Whereas emission of greenhouse gasses, especially by developed countries, is regarded as the primary driver of CC, population increase in less developed countries is considered the main underlying driver of LULC change (Farauta et al. 2012; Bone et al. 2017; Gondwe et al. 2019). Population increase alone might not lead to LULC change, especially in the developed countries, with diverse sources of livelihoods; it is only an issue in developing countries where the people are dependent on natural resources with less advanced agricultural technologies (Nkonya et al. 2011). Other drivers are economic and technological developments (Turner et al. 1995) and urbanization (Alemayehu et al. 2009). The desire to satisfy the insatiable human needs for a better life has impacted the environment, water, and livelihoods (Agarwal et al. 2000; Rawat and Kumar 2015). Hence, LULC change and CC are reoccurring without abatement unless drastic actions are taken (Mustard et al. 2005).

There is a synergetic relationship between LULC changes and CC with other factors causing impacts in our environment (Agyemang 2012; Vu et al. 2014). Whereas LULC change can lead to loss of soils and nutrients from poor agricultural activities (Fatumah et al. 2020), it is the CC that is responsible for transporting the sediments and nutrients to the receiving water bodies through river/stream flows, run-off, and shallow groundwater (Urama and Ozor 2010; Huong and Pathirana 2013). LULC changes and CC have been studied at global, regional, and national levels. Nevertheless, studying at the local level is essential to properly understand the drivers and impacts of these changes on livelihoods, land, and water. This is important since understanding human-nature interactions require explicit local knowledge and involvement of the local communities (Bremner et al. 2010; Wantzen et al. 2019).

Different ways have been used to identify LULC changes, including geographical information systems (GIS) and remote sensing using satellite data (Sun et al. 2013; Bu et al. 2014). However, the use of the Driver, Pressure, State, Impact, and State (DPSIR) model to link socio-economic growth effects on the environment (Kelble et al. 2013) has also gained popularity. It effectively describes the cause-effect associations between human-led development sectors and the environment (Pinto et al. 2013; Hou et al. 2014) and links its component elements (Dzoga et al. 2020). For example, the relation between the 'impacts' of a change in ecosystem or humans and the 'state' of the ecosystem depends on the system's threshold and carrying capacity (Kristensen 2004). The 'model's credibility is on its ability to serialize human effects on the environment, from drivers to the responses (Sun et al. 2016), and for establishing information on the status of the environment (Elliott 2002). Thus, it is an essential tool for decision-makers, policy-makers, water and land managers, and the general public for effective and sustainable management (Timmerman et al. 2011; Sekovski et al. 2012), at regional and local levels (Kelble et al. 2013; Sun et al. 2016). It has been applied in different circumstances, for example, to support fish and fisheries management in Lake Tana, Ethiopia (Gebremedhin et al. 2018); and to interpret the rate of siltation of water reservoirs in Mexico (Porta and Claret 2011). Unlike the remote sensing approach, the model allows researchers to interact with communities, identifying local drivers, pressures, states, impacts, and responses/coping mechanisms. Therefore, it provides a platform where local community knowledge can be incorporated into the scientific aspects of particular natural resources, bridging the gap between science and management and policy developments/reviews (Gebremedhin et al. 2018). Despite the value of information that the DPSIR model generates, valid for management and decision making, developed countries have applied it more (Gabrielsen and Bosch 2003; OECD 2003; Martins et al. 2012) than developing ones, especially those in Africa where most published papers are reviews (Agyemang et al. 2007; Gessesew 2017; Gebremedhin et al. 2018), including Uganda.

The objective of this study was to use the DPSIR model to identify drivers, and impacts of both LULC change and CC on the environment, water, and livelihoods in the L. Kyoga basin, using indigenous knowledge and stakeholder interactions. The research used focused group discussions (FGDs) and key informant interviews (KIIs) with the local community members, stakeholders, and field observations to identify these drivers, impacts, and coping mechanisms. The outcome of this research is the development of holistic and efficient LULC change and CC management approaches that are "bottom-up" driven, i.e., from communities to government policy and decision-makers (Bell and Morse 2001). This is in contrast to common top-down approaches in Uganda, where government through decision and policy-makers prefer to use expert, scientific/reductionist legislation and regulations with less consultation with local communities, which often leads to counterproductive efforts in dealing with LULC change and CC impacts (Wantzen et al. 2019; Kosamu et al. 2022). Most research studies have shown that both approaches are ineffective in dealing with the impacts independently (Hägerstrand 2001; Grêt-Regamey et al. 2013; Ricaurte et al. 2014; Wantzen et al. 2019). Whereas top-down approaches are useful at global, regional, and national levels, they do not emphasize intricate local resource user community perspectives and are problematic for the local communities to follow and implement (Reed et al. 2006; Gray et al. 2014). Bottom-up approaches are useful at the local community level and have been referred to as participatory or conversational (Bell and Morse 2001). This approach recognizes that the local communities are important in designing sustainable management measures as they determine what they will adjust to and when (Gray et al. 2014). Therefore, their views must be taken into consideration by top-down legislation and regulation if sustainable management has to succeed (Palmer et al. 2014). Therefore, the new paradigm shift, which this study is focused on, is towards the integration of both approaches (Reed et al. 2006). The findings are also applicable in the monitoring and implementation of United Nations Sustainable Development Goals (SDGs) in Uganda, which advocate poverty reduction (Goal 1), maintenance of clean water quality and sanitation (Goal 6), and taking corrective action on climate (Goal 13) by 2030.

## Materials and methods

### Study area

**Fig. 1:**
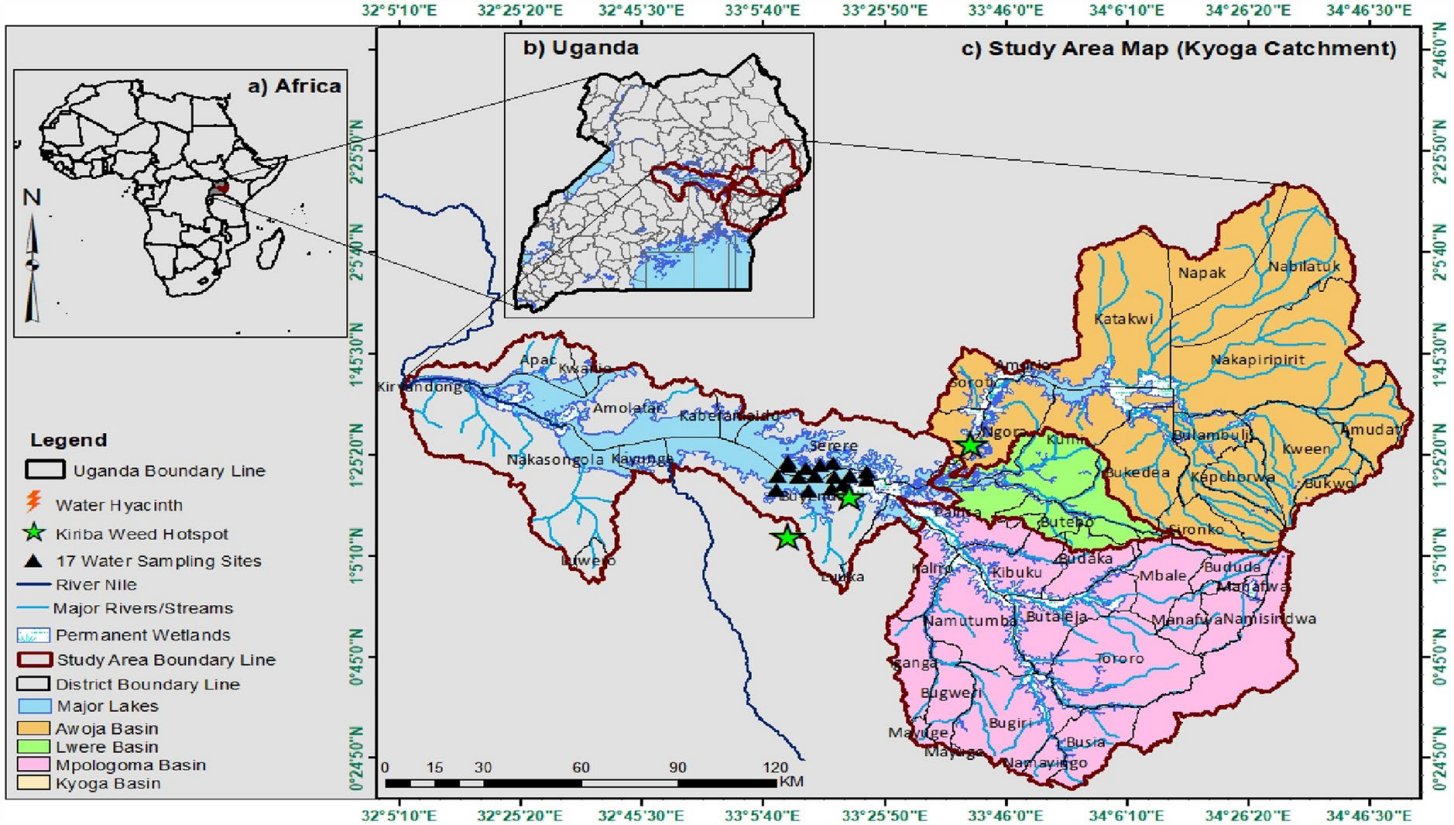
The location of the study area

The study area is in eastern Uganda, north of L. Victoria, with coordinates 0^o^24.50'N – 2^o^25.50' N; 32^o^5′10' E – 34^o^46′30' E, and covers three catchments, including Awoja, Mpologoma, and Lwere, (Fig. 1). These catchments cover a more significant part of the eastern catchments of L. Kyoga, which is a recipient of the water from these catchments. Lwere is the smallest of these catchments, with an area of 1500 km^2^; Mpologoma is the second largest in the basin with 9,000km^2^, with 13 sub-catchments (MWE 2018). Awoja is the most extensive catchment of the basin, with an area of 11,000 km^2^ and 14 sub-catchments (MWE 2015). The study area covers 34 of the 58 districts of the L. Kyoga basin. Its population estimate is 9.4 million people, with the youth below 30 years constituting over 75% of this population (UNBS 2020). The study area has Mt Elgon, 4,321 m above sea level (m.a.s.l), at the border between Uganda and Kenya. This mountain is the source of rivers and streams that drain the catchment and is also a settlement, agricultural, and forestry area, hence playing a significant role in LULC changes and CC. This mountain and lakes in the area, like Kyoga and Victoria, are the leading influencers of the climate of the study area (Camberlin 2009; MWE 2018). The two phenomena of LULC change and CC are the most critical factors affecting this basin, impacting livelihoods and the environment.

**Fig. 2:**
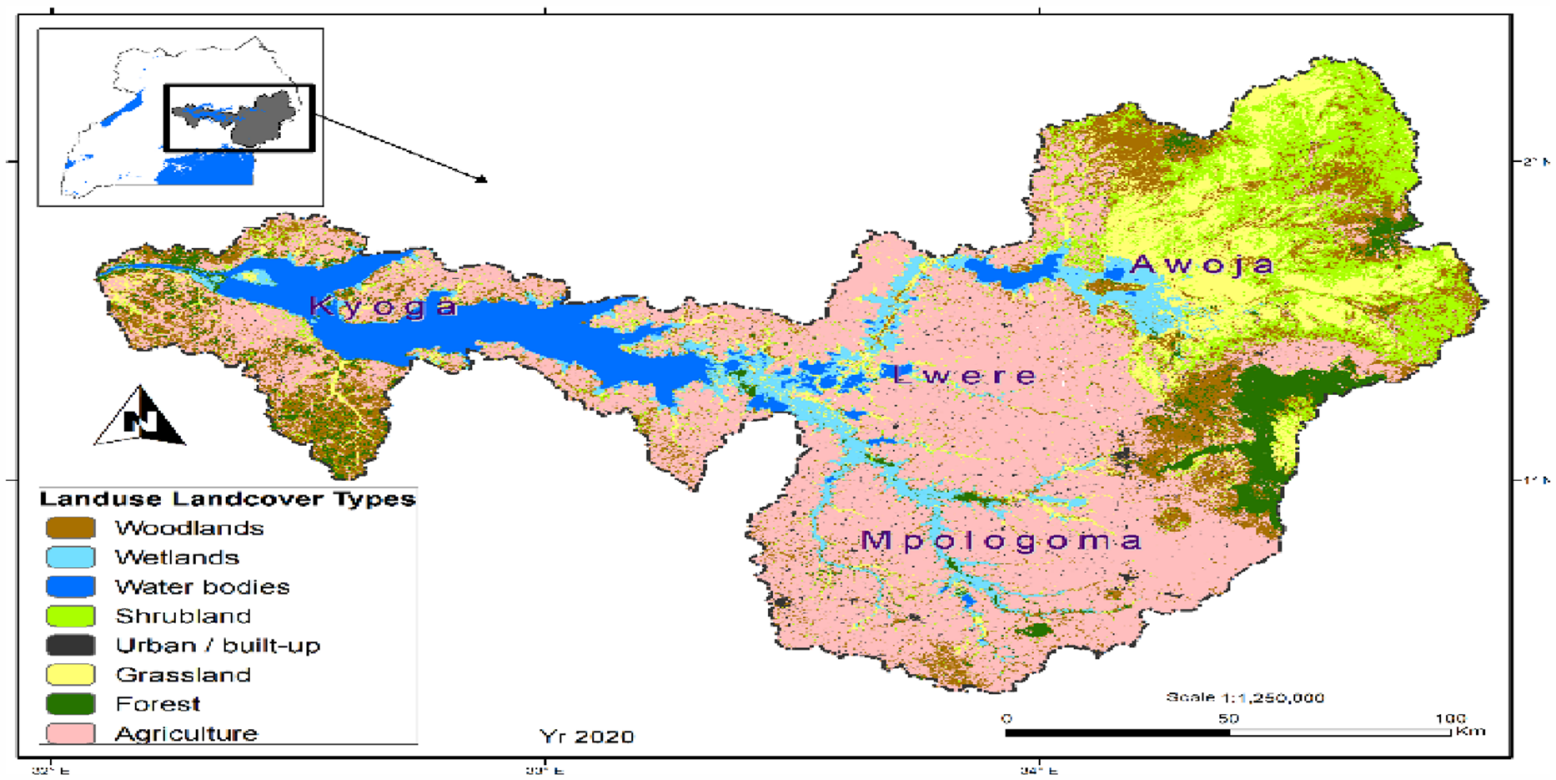
The current LULC map of the study area ( Source: Obubu et al. 2021a)

The study area was divided into three segments, including (1) the upstream, which covered the top and slopes of mountains Elgon and Kadam, the districts here included Kapchorwa, Nakapiripirit, Bududa, Bukwo, and Kween (2) the mid-stream, which included the lower slopes of Mt. Elgon and the higher grounds, districts covered here included Mbale, and Kumi, Butebo; and (3) the downstream, which included the flood plains of Teso and parts of Busoga. Districts here included Serere, Pallisa, Ngora Butaleja, Buyende. Upstream districts experience erosion due to the steep nature of the land, with bare land as a result. Mid-stream areas were observed with lush wetlands due to nutrient and sediment deposition from the upstream, resulting in fertile agricultural soils. The downstream areas had extensive wetlands, as the river flow slowed down before discharging water into L. Kyoga. Further deposition of sediments occurs here, making soils and wetlands nutrient-rich; hence they are highly cultivated and degraded (Fig. 2). The 2020 LULC map was developed using remotely sensed data from global optical satellites, obtained from the United States Geological Survey (USGS) (https://earthexplorer.usgs.gov), accessed on 20^th^ November 2020 as described in Obubu et al. (2021a). Agriculture was the main LULC change activity in the study area (Fig. 2). The cultivation diminishes the ecological buffering role of wetlands, including the uptake and storage of excess nutrients and filtering sediments.

The topographical division influences the state and impacts of LULC change and CC under the same drivers. The study area also had two distinct climatic zones; the drier northern cattle corridors of Karamoja and parts of Teso, which receive less rainfall, and the Mt. Elgon area, which receives high amounts of rainfall, making the region vulnerable to impacts of CC and LULC change (Mbogga 2012; MWE 2013). The main LULC activities in the study area include converting land, forests, and wetlands into agriculture, mainly for rice fields, human settlement, and urbanization. Further, sand mining, quarrying for marram, and artisanal mining of minerals, such as limestone, occur in the area. Land use, land cover changes, and CC were established through FGDs and KIIs with the local communities. The LULC changes, alongside CC impacts, primarily result in floods, landslides, mudslides, and droughts, adversely affecting the environment, water, and livelihoods over time.

### Data collection and analysis

#### Data collection

**Fig. 3:**
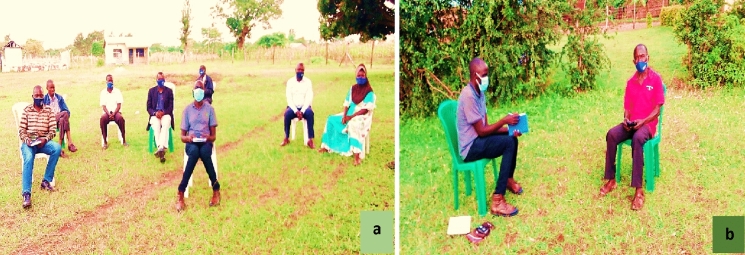
A section of a FGD **a** and KII **b** in Busiu Mbale

Data collection was done using three principal approaches: focus group discussions (FGDs), key informant interviews (KIIs), and field observations. The FGDs is a key that was first developed in the 1920s (Morgan 1998), formalized in the 1940s (Madriz 2000), and has been refined and widely used by various scientists for qualitative data collection (Onwegbuzie et al. 2009; Nyumba et al. 2018). The data collection was done in representative districts in the upstream, midstream, and downstream areas to capture all study areas' perceptions and views. Focus groups consisted of a minimum of 12 and a maximum of 15 members, although there were 21 members in Kumi district. Fifteen members for each FGD have been recommended and used by many authors to allow members to express themselves (Onwegbuzie et al. 2009; Makwinja et al. 2021), but at the time of this study, it allowed FGDs to follow standard operation procedures (SOPs) against Covid-19 pandemic, which included social distancing and wearing of facemasks (Fig. 3).

The FGDs and KIIs took a maximum of one and a half hours and were moderated by two people, the researcher, and a well-trained assistant, to ensure consistency, as recommended by Maxwell (2012). There were nine FGDs, and thirteen KIIs carried out in the study area. That number was reached as information saturation was attained, following Sandelowski (2008). The FGDs were organized with the help of the Ministry of Water and Environment (MWE) deconcentrated regional office, Kyoga Water Management Zone (KWMZ), in conjunction with district community development officers (DCDOs). Open-ended questions were administered on the types of LULC changes, CC, and also on the drivers, pressures, states, impacts, and responses to LULC changes and CC. The participants ranked the drivers and impacts according to the Linkert scale, from 1–5 (1 = most important, and 5 = least important) (Solehana et al. 2019; Tien and Thuy 2020). KIIs followed a phenomenological tactic as described by Patton (1990). The KIs were elderly, above 55 years of age, and residents of the study area. Others were experienced office bearers rendering service to the community, from government and non-government agencies. The independent data and information from KIs were used to validate the information from the FGDs. All FGDs and KIIs strictly followed ethical guidelines recommended for data collection (Levy and Lemeshow 2013).

Field observations by the researcher during the time of data collection were also used to collect the current information on the drivers and impacts of LULC changes and CC in the study area. Here, the state of wetland degradation and conversion into rice fields was observed, and forest degradation, among others, was observed firsthand during data collection. The exercise took over three weeks in total but was taken in three segments. Degradation activities like conversion into rice growing were observed directly, and pictures were taken (Fig. 5), as reported in similar studies in Zimbabwe and India (Crecious and Lazarus 2013; Das and Basu 2020).

Literature reviews were also carried out to obtain relevant data and information for the study. For example, the Uganda Bureau of Statistics (UBOS, www.ubos.org), accessed on the 25th May 2021) was visited to obtain accurate and projected population data. Secondary data on the river discharges, water levels, and water quality for Mpologoma, Awoja rivers, and L. Kyoga were obtained from MWE.

### Data analyses

FGD and KII data were analyzed through coding and organization into thematic groups for easy presentation. This was done following the constant comparison analysis approach (Onwegbuzie et al. 2009), which was first developed by Glaser et al. (1968). Data were then grouped into drivers, pressures, state impact, and responses. Water discharge data, water level, and water quality data were analyzed in Excel and graphs and developed into tables.

## Results

**Fig. 4:**
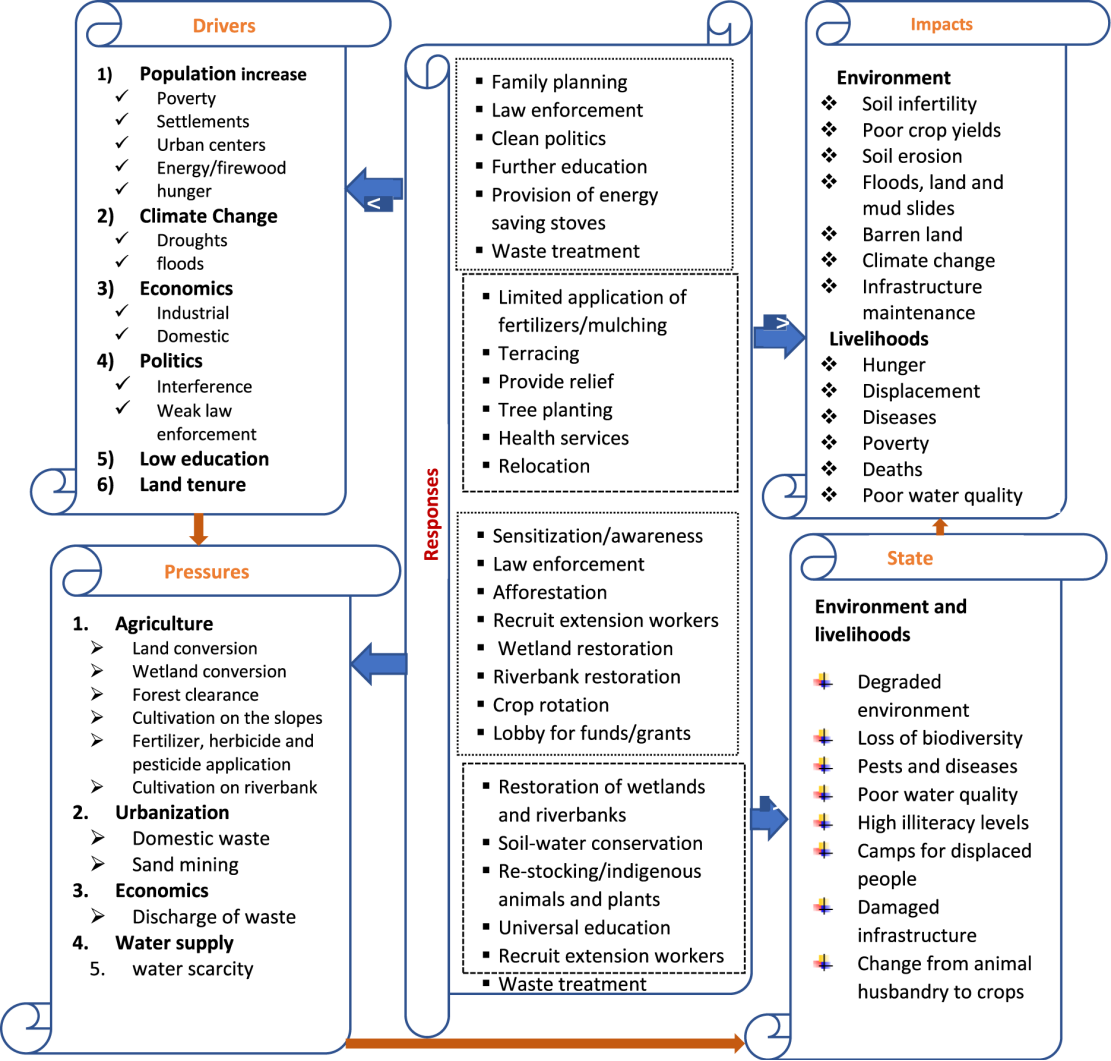
DPSIR framework model of the findings of LULC change and CC in the study area

The results obtained from the FGDs and KII are summarized in the DPSIR conceptual framework model (Fig. 4).

## Discussion

### Drivers (D) of land use, land cover, and climate change

Drivers are essential societal needs like food, shelter, energy, and transport that have to be met regularly, and are in two categories, namely proximate (Dregne 2002; Lambin et al. 2008) and underlying (Tiffen et al. 1994; Kabubo-Mariara 2007). The requirements for meeting these needs vary from one country to another and from urban to rural settings. In less developed countries, like Uganda, especially in rural settings, the immediate needs are met mainly by using natural resources (Reynolds et al. 2007; Bremner et al. 2010). During data collection, six significant drivers were identified by the local communities. These included population increase (Fig. 4), climate change, economic development, politics, low education levels, and land tenure. However, these drivers do not work in isolation, but rather synergize with one another to cause impacts on land, water, and livelihoods.

**Fig. 5:**
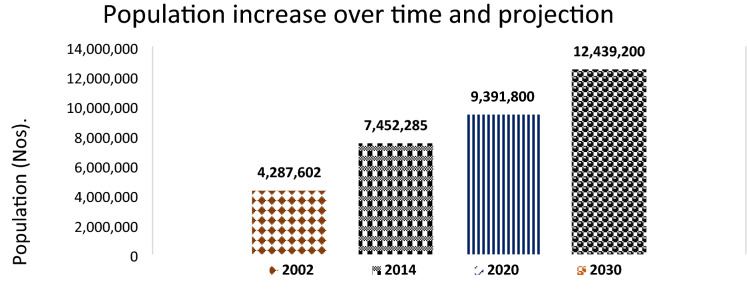
Population dynamics over the study period (2000–2020) and projections for 2030

The population increase was identified as a number one driver in eight of the nine FGDs carried out, except in Nakapiripirit (Karamoja region), where low education levels/high illiteracy rates ranked highest. Population pressure has impacted land, wetlands, and forests, which have been degraded to meet the essential human needs, primarily through agriculture. UBOS data indicates the study area as one of the most populated regions in the country (UBOS, 2020), as shown in Fig. 5.

Whereas high population impacts LULC change and CC, the youthful population structure in Uganda and in the study area in particular, where up to 78% of the population is below 30 years of age, is worrying. Uganda's average population density is estimated at 228/Km^2^, with a growth rate of 3.32% and a fertility rate of 4.78 births per woman, ranking among the highest globally UBOS (2020). Such population growth rate is associated with rapid urbanization and high poverty levels observed in the study area. With an unemployment rate of over 2.9%, which is higher than the national rate of 2.44, this population is now heavily dependent on natural resources as sources of income or doing petty jobs in the urban centers. Therefore, the young, energetic population, dependent on parents, has become a real burden to the natural resources, as they are being used as human labor to degrade natural resources, which in turn has resulted in CC, as has been observed by other studies (Reynolds et al. 2007; de Sherbinin et al. 2008; Alemayehu et al. 2009; Bremner et al. 2010; Akhtar et al. 2011; Kirui and Mirzabaev 2015; Ashfaq et al. 2019).

Population-related drivers also act synergistically with other community aspects, like poverty, socio-economics, and environmental factors, causing LULC change and CC. Poverty, an underlying driver, in synergy with other factors was the second driver of LULC changes and CC, as poor people depend on natural resources, as pointed out by other authors in Kenya (Kirui and Mirzabaev 2015), the USA (Bremner et al. 2010) and Germany, (Bisaro et al. 2014). National poverty indices showed Eastern alongside the Northern regions as one of the poorest in Uganda (UBOS 2020; UNICEF 2020).

The need for shelters, energy in the form of firewood and charcoal and hunger, low education levels, weak law enforcement, urbanization for alternative income, lack of cash crops, loss of cultural values, and high cost of land were other drivers of LULC change and CC; all these drivers are related to the high youthful population. This agrees with what was found by other authors (Lambin et al. 2001; Geist and Lambin 2002; Harte 2007). In Nakapiripirit and Karamoja areas, low education level, hence high illiteracy, was the number one driver. Production of domestic and industrial effluents from Karamoja mineral mining and domestic effluents from urban centers, including Mbale, and Soroti cities, Iganga, and Tororo municipalities impacted the land and water resources as pointed out by other studies (Kristensen 2004; Anaba et al. 2016). Climate change was also identified as one of the significant drivers of LULC changes in the study area. This was in agreement with the findings of CC in the study area, reported in the related study (Obubu et al. 2021a). For example, high population growth, led to establishment of urban centers, with economic activities, and increased demand for energy and construction materials. These in turn resulted in LULC change in form of deforestation and wetland degradation, hence increase in CC; meanwhile, CC leads to forest and wetland loss, a vicious cycle that is observed globally (Bremner et al. 2010; Nkonya et al. 2011; Bisaro et al. 2014). Political interference was cited as one of the drivers of LULC changes, especially in wetland and forest degradation. This was cited in Pallisa, Kibuku, Kumi, and other districts, where politicians would urge their constituents to defy regulations and encroach on wetlands and forests for political gain, especially during elections. Limoto wetland, located between Pallisa and Kibuku is an example where political interference seriously compromised conservation activities.

Land tenure systems were drivers of LULC change and CC. There were four types of land tenure systems in the study area: customary ownership the most dominant type, community; leasehold in urban centers; and cultural land ownerships. Customary tenure was identified as a driver of LULC change as parents have to keep on sub-dividing the finite land to the infinite number of children over generations. This would result in land fragmentation and conflicts between families. It further demonstrated the connection and synergy between different drivers (population and land tenure). This study revealed that, in Bududa, the amount of land a son inherits is dependent on how well-behaved his wife is to the parents-in-law. The participants noted the difficulty in commercializing customary, communal, and cultural lands without the parents' permission, community and cultural leaders, and the result was land fragmentation for agriculture and settlement. In Nakapiripirit, the communities accused the local government leaders of taking advantage of communal land tenure by selling and leasing it to commercial developers like companies and academic institutions without consulting them, thus changing 'LU's purpose. Acerer village and Nakapiripirit town council were cited as examples where such abuse of land tenure systems was rampant. These findings agree with what was found in Kenya by Kakubo-Mariana (2007), where land rights and land conservation issues were discussed.

### Pressures of land use, land cover changes, and climate change

The need to meet the drivers of LULC change and CC results in exploiting natural resources; these exploitative activities place pressure on the environment (land and water) (Kelble et al. 2013). The pressures were grouped into thematic areas, with agriculture, urbanization, and economic development identified and leading pressures (Fig. 4). Many people are entirely dependent on agriculture, just like it is the mainstay in the country, where it supports over 70% of the local economy (Ojara et al. 2020). Although maize, sorghum, rice, and millet were cultivated across the study area, Arabica coffee was unique to the mid and upstream districts on Mt. Elgon like Mbale, Bududa, Kapchorwa, as the weather here and high altitudes above 1600 m.a.s.l favors it (MWE 2015). Cassava was dominant in the downstream districts of Serere, Kumi, and Pallisa. The demand for food resulted in the conversion of wetlands, riverbanks, l, and forests into agricultural lands as observed in Limoto wetland in Palissa, a situation observed in Kampala, Uganda (Matagi 2002), in Kenya, Malawi, and Tanzania (Kirui and Mirzabaev 2015). There was also reported cultivation on the steep slopes of Mt. Elgon with minimum terracing. This practice loosened the soil, hence resulting in mudslides and landslides during rainy seasons. These damaged infrastructures and caused deaths annually, as was also pointed out by Dregne (2002).

Boosting agricultural production to meet food demands is now supported by applying agrochemicals, which included fertilizers (NPK, Organic manure, Urea, DAP, CAN, TAP, Allwin gold super, Vegimax, Evergreen and Aminocop), pesticides (Super grow, Dudu cipher, Rocket, Stricker, Tafgor/dimethoate, Ambush, Dudu maki, and Golden drop), and Herbicides/Fungicides (Roundup, weed master, (2-4-D) Diamine, Force up and Ametrine) as reported by the participants. The participants emphasized that, without their use, the farmers get poor and low yields. Other reasons for using these chemicals included loss of soil fertility, losses due to pests and diseases, and CC. It was only in Nakapiripirit, where agrochemicals were not used yet, due to land availability, small population, good soil fertility, and nomadic lifestyle.

The communities started using these chemicals at different times; the upstream districts over 30 years ago, while the downstream districts started as recently as five years ago. This confirmed the argument that upstream districts lost fertility earlier due to soil erosion, while the downstream district had fertile soils due to deposition of nutrients and sediments from upstream districts. Land fragmentation has affected most parts of the study area, as shown by the widespread use of agrochemicals, except in Nakapiripirit. The KIs indeed confirmed that land in Nakapiripirit was plenty and fertile in the past 30–40 years. Fallowing eliminated the need for the use of agrochemicals. These agrochemicals affect soils, but especially the water quality of the receiving water resources, like rivers Awoja and Mpologoma and L. Kyoga, through run-off, facilitated by CC (Obubu et al. 2021a). Similar observations were made in Qatar and Ethiopia (Akhtar et al. 2011; Gebremedhin et al. 2018).

Urban centers have put pressure on land as agricultural land, forests, and in some cases, wetlands have been converted into urban centers, like Imatakojo forest in Pallisa district, which was converted into an urban center, as has also been observed in other areas (McKinney 2002; Tang et al. 2005; Akhtar et al. 2011; Rawat and Kumar 2015; Gebremedhin et al. 2018). Forests have been degraded to provide timber for construction and energy in the form of charcoal and firewood. Meanwhile, domestic wastes from these centers are also sources of contamination of land and water resources. Sewerage effluent data from Soroti and Mbale cities and Tororo and Iganga municipalities were obtained from the MWE from 2014 to 2021. The mean annual values for Electrical conductivity (EC), pH range, Total Suspended Solids (TSS), Total Phosphorus (TP), Total Nitrogen (TN), and Biochemical Oxygen Demand (BOD) were compared with the National Environment (Standards for discharge of effluent into water or land) Regulations 2020, (Table 1).

**Table 1:** Domestic effluents against wastewater discharge standards

Year	EC (µS/cm)	pH (pH units)	TSS (mg/l)	TP (mg/l)	TN (mg/l)	BOD (mg/l)	COD (mg/l)
2014	884	8.0–10.0		3.16		35	231
2015	612	7.1–9.2	75			12	144
2016	1039	6.8–9.4	136	3.6		187	1438
2017	837	6.8–8.6	210			18	152
2018	1098	6.9–9.1			75.7	7	264
2020		6.8–9.9				69	156
2021	1059	5.3–11.0	309	7.9	52.8	26	366
Effluent Standard	750	5.0–8.5	50	5	10	50	70

Data source: Ministry of Water and Environment

All the parameters were at least above the standards in different years, putting pressure on the water quality of L. Kyoga and land. The EC met the standards in 2016, and pH tended to alkalinity in all the years. TSS, COD, and TN did not meet the standards; BOD in two years (2016 and 2020) out of the seven years did not meet the standards. The result is the pollution of water resources and land. According to local communities, other economic activities in the study area that exerted pressure on natural resources were the development of agro-processing industries like distilleries, where raw hot waste was released into the environment without treatment, affecting receiving water ecosystems such as River Nakibiso in Mbale district. As a result, parts of the river had been seriously contaminated, rendering the water unfit for any use. The participants in Nakapiripirit district also reported pollution from artisanal mining activities of minerals like gold. Meanwhile, most industries in Mbale and Tororo districts operated dry processes, hence the need to monitor gaseous emissions and their effects on the atmosphere and CC, which was not done in the study.

### State of land use, land cover change, and climate change

Given that drivers are the human needs, and pressures are the activities carried out using the natural resources to satisfy the needs, the transformation of natural resources, hence the loss of quality ecosystem services they provide, represents the state of particular natural resources. (Kristensen 2004; Gessesew 2017).

#### State of water and environment

Participants ranked land, wetlands, forests, and riverbanks as the most degraded land use, especially for agriculture. Soil erosion, bare land, and formation of gullies, especially in the upstream districts at the slopes of Mt. Elgon, but also in Pallisa was, reported. Protected areas are getting degraded by displaced people as the population keeps increasing. There was increase in pests and diseases, which could not be independently verified due to a shortage of data. One of the salient issues discovered by this research was the conversion of the most revered cultural land/sites into agriculture, especially in Pallisa, Bududa, and Nakapiripirit districts. Some of these cultural sites included Nashinda, Nashoba, Yerakha, Namasho, Nameremu, and Nehoyo in Bududa, which were hitherto protected by the communities. Up to 30 years ago, they were sites of sacrifices and celebrations during catastrophes and in plenty. Indirectly, they became bushy and forested, thus refugia for biodiversity (wild animals and birds), reported in Burkina Faso, Benin, and Togo in Weste Africa (Juhe-Beaulaton 2006; Kokou and Sokpon 2006). Some elderly participants and KIs associated the current droughts, floods, and diseases with the encroachment to these sites as they believe the "gods" were "annoyed". Government and other stakeholders should take advantage of these cultural values and beliefs and integrate them into conservation policies and laws to protect the environment and natural resources in the study area.

**Fig. 6:**
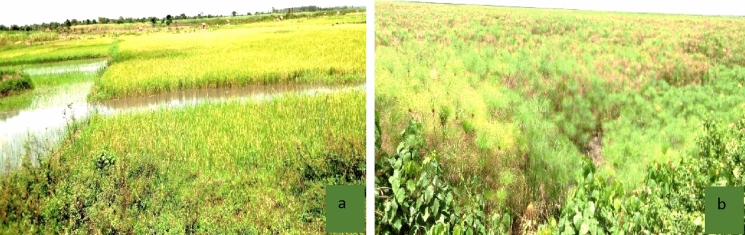
Parts of Mpologoma wetland, **a** converted for growing rice; and **b** intact wetland area

Field observations during data collection also confirmed the degradation of natural wetlands through rice growing (Fig. 6). These findings agree with the work done in the Namataba wetland (Namaalwa et al. 2013) and Naigombwa wetland (Were et al. 2020), which are parts of this study area.

Meanwhile, the National Water and Sewerage Corporation (NWSC), the water supply agency for Mbale City, reported increased siltation, sedimentation, and coloration of water in River Manafwa, an intake point, during wet seasons, due to soil erosion, especially from artisanal sand and iron mining in the upstream areas (Table 2).

**Table 2:** Seasonal variation of water quality in R. Manafwa (intake for Mbale water supply)

Season	pH	EC (µS/cm)	Turbidity (NTU)	Colour-app (PtCo)	TSS (mg/l)
Wet	7.1	113	1348	10,843	1900
Dry	7.2	171	475	5179	774

Data source: NWSC, Mbale district

There was a distinct variation in water quality parameters in wet seasons (March–May) and dry seasons (June to August), with the management reported an increase in the quantities of water treatment chemicals, potentially increasing the cost of water supplied to consumers. For example, the corporation used water treatment chemicals worth 5400 USD during the dry season compared to 8700 USD during the wet season. Indeed, some participants in this area reported that tap water supplied by NWSC was often brownish, especially during heavy rainy seasons.

There was also reported loss of biodiversity, especially wild animals like leopards, antelopes, baboons, and wild birds like eagles that dwelt in wetlands, forests, and woodlands, due to clearance of their habitats. These were pointed out by the KIs, who were knowledgeable about these animals before they disappeared.

#### State of livelihoods in the study area

Many people are being displaced internallyby floods, land, and mudslides. For example, over 500 people were resettled in Kiryadongo with a further 600 in Bulambuli. There were shortages of some basic needs, including sanitation, food, and water supply for domestic purposes. There was damage to homes and infrastructure including roads and water supply lines, affecting livelihoods. This shows that CC is acting synergistically with LULC changes, exacerbating the damage to the environment and negatively impacting human livelihoods through poor sanitation, shortage of food, and shelter. Further, participants, especially in the downstream districts of Serere, Kumi, and Pallisa, the traditional cattle-keeping communities, reported a reduction in domestic animals due to a shortage of grazing land.

### Impacts of LULC change and CC on the environment, water, and livelihoods

#### Impacts on land

Loss of soil fertility was reported across the study area as a number one impact of LULC change and CC. The participants reported low harvests compared to what they used to get from the exact acreages of land 30 years ago. Crop and animal yields were of poor quality due to low soil fertility, which agrees with findings by Osbahr et al. (2011) in Mbarara district, western Uganda. The local communities noted that soil erosion had eroded the topsoil especially in the steep slopes of Mt. Elgon, leaving infertile underground soil, which agrees with what was reported by other authors (Alemeyehu et al. 2009; Porta and Claret 2011; Anaba et al. 2016). Expect in Nakapiripirit where land is still fertile, the rest of the study are reported increased application of agrochemicals to boost agricultural production, which is in line with a study by Akhtar et al. (2011).

The land resources have been degraded and no longer support the population; hence the local communities are encroaching on protected lands, like forests and wetlands, for crop production. The clearance of forests and degradation of wetlands has resulted in erratic precipitation and droughts (climate change), and the vicious cycle is getting worse every year, as also reported by Bremner et al. (2010) and Obubu et al. (2021a). At the national level, forest coverage has reduced from 24% in the 1990s to 12.4% in 2020 (MWE 2020). Relatedly, wetland coverage has reduced from 15.6% in 1994 to 8.9% in 2020 (MWE 2020). The conversion to agricultural land is the main LULC change, as reported in the tropics (Geist and Lambin 2002). There was a loss of biodiversity in forests and wetlands.

Further, the poor-quality crops and animals are more vulnerable to infestation by pests and diseases, with participants reporting an increased outbreak of pests and diseases exacerbated by CC; this was also observed by Hisali et al. (2011). These observations were proved by the increasing use of pesticides over the last five years generally and over 30 years in the upstream district, which experienced the loss of soil fertility due to soil erosion due to steep terrain. There were also reported increased costs incurred by the respective local government authorities and members of communities in the maintenance, repair, and replacement of damaged infrastructure and facilities, including roads, homes, health centers, and schools.

#### Impacts of LULC change and climate change on livelihoods

There were displacements from land and mudslides and deaths in different communities. A total of 427 people were reportedly killed by floods, land, and mudslides from 2010 to 2021, with Bududa district the most affected (417 people), Nakapiripirit (6 people), Kapchorwa (3 people), and Mbale (2 people). There was also an increase in temperature, including warm nights, and mosquitoes due to CC, thus malaria in areas that used to be cool, e.g., Kapchorwa, as reported in a related study (Obubu et al. 2021a). There were increased conflicts within communities and between communities and law enforcement agencies like National Forest Authority (NFA) National Environment Management Authority (NEMA) over natural resources like wetlands, fertile land, and forests; for example, Limoto wetland in the border between Pallisa and Kibuku districts. These conflicts were also reported in the fishing communities in Lake Tana, Ethiopia (Gebremedhin et al. 2018), Uganda (Hisali et al. 2011; MWE 2015), and in Southeast Asia (Francisco 2008). Measures should be undertaken to ensure that conservation and sustainable resource utilization co-exist.

There was increased poverty and hunger among the local communities as agriculture was not adequately productive due to floods, [poor soils, and droughts. Lack of not only cash crops but also markets for agricultural produce was affecting family income. Poverty and hunger resulted into school dropouts, child labor, early child marriages, and pregnancies, and gender-based violence, where women reported husbands abandoning homes, leaving women with the burden of raising children, as was also observed in Ethiopia (Gessesew 2017), and other developing countries (Bremner et al. 2010). Participants reported increased disease outbreaks, including malaria and trachoma, with some claiming that even the covid-19 pandemic could be related to CC.

#### Impacts of LULC change and climate change on water resources

**Fig. 7:**
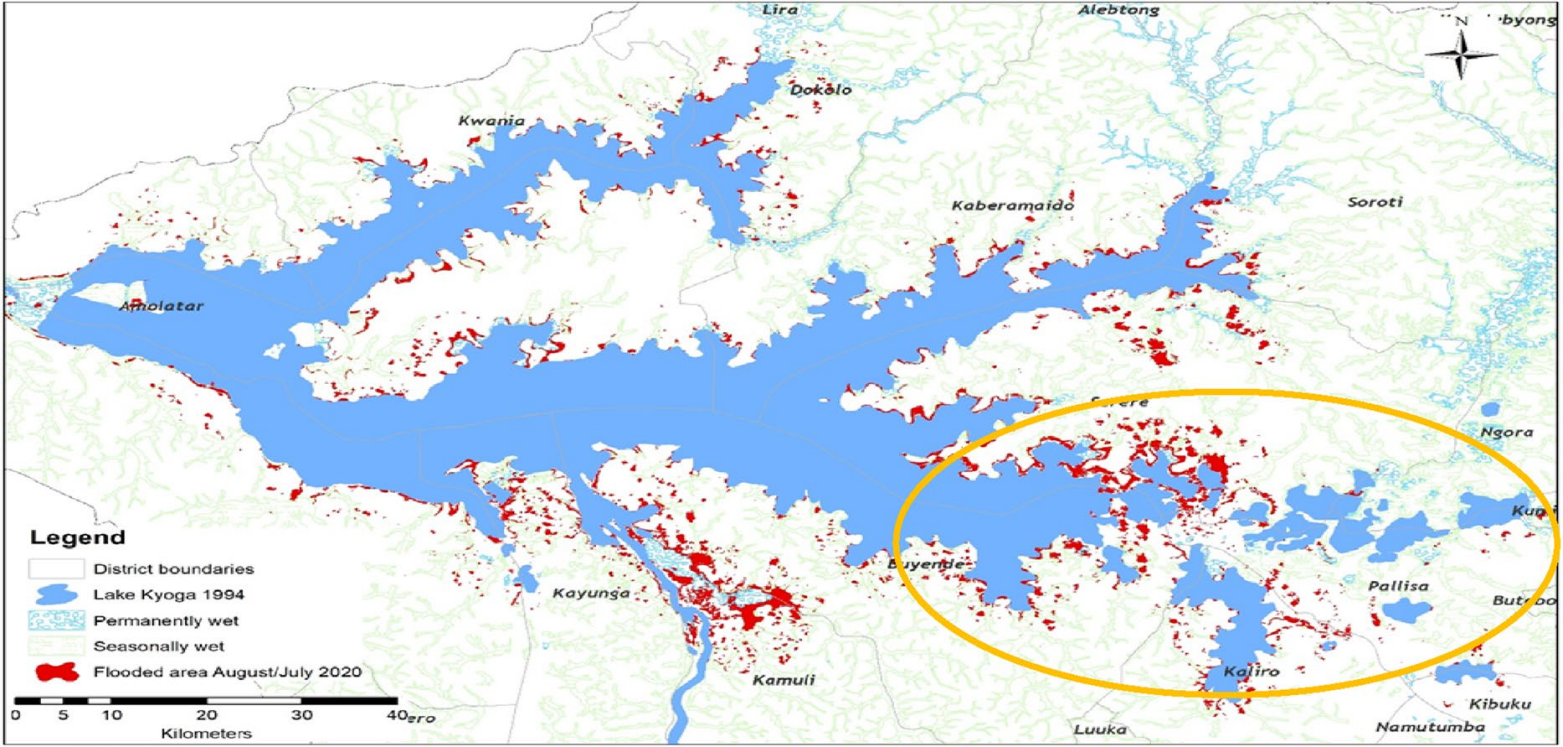
A map showing the extent of 2020 floods in L. Kyoga, the yellow circle shows the study area. Data source (MWE)

When asked about the impacts of LULC change and CC on the water resources, especially Lake Kyoga, participants from Serere, Kumi, and Pallisa districts near the lake, pointed out several impacts. For example, flooding at the lake shores, especially following the heavy rains, was more frequent than 40 years ago. Indeed, the years 2007 and 2020 had the most damaging floods, with the floods of the year 2020 leading to the breaking of the water level record of 1964 (Table 3). Data obtained from MWE was used to map the 2020 floods to show the extent of spread and was found to support the local communities' observation (Fig. 7). The analysis showed that the spatial extent of the lake expanded by 12.2%.

**Table 3:** Record-breaking water level trends for major Ugandan lakes

Water levels (m)				
SN	Lakes	1964 (Old record)	2020 (New record)	Difference
1	Kyoga	13.25	14.41	1.16
2	Victoria	13.41	13.48	0.07
3	Albert	14.2	14.68	0.48

Data source: MWE

The 2020 floods did not affect only L. Kyoga but other major lakes in the country. A summary of the water levels of three major lakes in Uganda is given in Table 3.

**Fig. 8:**
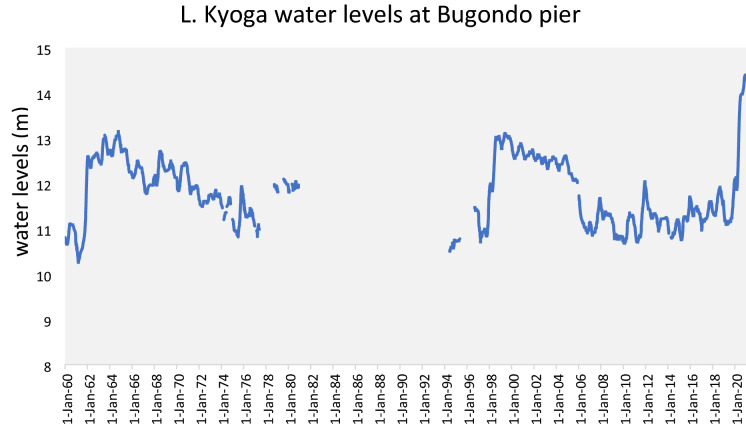
Water level trends for Lake Kyoga, over six decades. Data source (MWE)

Lake Kyoga was the most affected by these floods, as pointed out by the participants during the FGDs and KIIs, which agreed with the data from the MWE (see Table 3 and Fig. 8). The water level data gaps in the 1980–1990s were caused by the insurgency, which interrupted data collection. Lakes Kyoga and Victoria reached new record levels in May 2020; meanwhile, L. Albert, located downstream of the two lakes, broke the record in October 2020.

The record-breaking floods could be associated with climate change in the study area, which has been steadily increasing in the last two decades (Obubu et al. 2021a). Floods are responsible for transporting and loading nutrients and sediments from the catchment into the lake, as reported in the related study (Obubu et al. 2021b). This showed the connection between LULC changes and CC and why these two components should be studied together. They were also responsible for the flushing out and drowning of *S. molesta*, an invasive weed that had invaded L. Kyoga. The participants and the literature reviewed on the floods in L. Kyoga agreed on the damages caused by floods on people, infrastructure, animals, and crops, which resulted in hunger, poverty, and malnutrition, especially among children. They also affected fishing by dislodging shoreline vegetation into floating suds, which blocked landing sites, interfered with boat landing and leaving, and water supply to the riparian communities as observed in Mugarama and Iyingo landing sites, in Serere and Buyende districts, respectively. This was also observed in other parts of the world (Keskinen et al. 2010; Kundzewicz et al. 2014). The fishing community also reported a reduction in fish catches, destruction of fish breeding sites, increase in siltation of the lake and wetlands, poor water quality, increased risks of attack from reptiles like crocodiles, snakes, and monitor lizards. Further, the area witnessed a higher abundance of mosquitoes, with increased incidences of malaria, according to participants.

Application of agrochemicals in the catchment and soil erosion contributes to pollution of Lake Kyoga and the observed brown water in the rivers laden with sediments, as has been pointed out by other authors (Ogutu-Ohwayo et al. 2013; Andama et al. 2017). On the other side, floods helped drown and flush out the invasive weed, *Salvinia molesta* from L. Kyoga, although it also helped spread it into the catchment and could indeed lead to its reinvasion later, as reported in other studies (Oliver 1993; McFarland et al. 2004; Lal 2016). The FGDs confirmed that the local communities had rich knowledge and experience of environmental issues. This presents an opportunity for engaging the community members in environmental protection and conservation practices.

Pollution of L. Kyoga has exposed it to invasion by invasive water weeds like *S. molesta*. When the participants, however, were asked why L. Kyoga was invaded by invasive water weeds, especially *S. molesta* and water hyacinth; there were varied responses. Some participants thought minister Nankabirwa brought it; others said it was from L. Victoria. At the same time, some pointed out nutrient enrichment from the catchments, warm water, many bays where the weeds are protected were responsible. Other contributing factors included slow water flow and others said it was the act of 'God' These responses were similar to the scientific findings (Ogutu-Owahyo et al. 2013; Wanda et al. 2015; Andama et al. 2017), although the local community knowledge showed more details, which are often missed when only scientific approaches are used. This research shows the importance of integrating scientific knowledge with social or community-based perceptions, observations, and experience to identify causative factors for environmental degradation, hence developing more reliable and lasting sustainable solutions to the environmental and CC issues. The participants listed consequences of invasion by the weeds, including fish kills, reduced oxygen, and difficulty in navigation.

On the massive fish kills, especially of Nile perch in Lake Kyoga in 2020, the participants outlined the possible causes as pesticides in household sprays and from the gardens in the catchments drained to the lake through floods, increased heat in the water, Nile perch diseases, and poison fishing was. Meanwhile, the joint report on the massive death of Nile perch issued by the Ministry of Agriculture, Animal Industry and Fisheries (MAAIF 2020) pointed out reduced dissolved oxygen levels. The reduction in dissolved oxygen could have been caused by deposits of organic matter drained by the floods and drowned *S. molesta.* The decomposition of these organic matter by aerobic bacteria could have resulted in the reduction of dissolved oxygen, hence the death of Nile perch, which is sensitive to low oxygen levels of less than 2.5 mg/l (hypoxia condition) (Goudswaard et al. 2011). Both the community and scientific views are in consonance, hence are essential for management purposes.

#### Linkage of LULC change and CC from catchments to the lake

One of the main objectives of this social part of the research was to find out whether the local communities were aware that LULC change activities in the catchment and CC impacted the water quality of rivers and L. Kyoga. When the question of connectedness was posed to the FGDs and KIIs, the response was a resounding no. The participants in all groups, from the upstream, mid, and downstream, even those at the landing sites, were unaware that catchment activities were impacting the water quality of Lake Kyoga. As a result, the researcher educated the participants on the relationship between LULC changes and CC in the catchment and their adverse impact on water ecosystems. This finding exposed a perception gap in the connectivity between land and water and calls for awareness of the linkage. There was, therefore, a strong recommendation by participants on sensitization of the local communities on this matter. The issue of LULC change activities in the catchments being responsible for the water quality of the receiving water bodies has been well researched and proven elsewhere (Donohue et al. 2006; Miranda et al. 2014), but are still a challenge in Uganda. These findings illustrate the importance of integrating scientific and local knowledge to manage natural resources better. The participants requested feedback on the research findings and increased the extent of sensitization.

### Responses to LULC change and climate change impacts

This component of the DPSIR has responses from different sections of society, including local community coping mechanisms central and local government responses.

#### Local community coping mechanisms

The participants gave a list of coping mechanisms to alleviate the impacts of LULC changes and CC, shaped by prolonged exposure to these impacts. These included moving to urban centers for business purposes since the land was too fragmented for agriculture. They use agrochemicals to improve crop and animal production, plant tree to combat CC impacts, and to meet timber and firewood demands. For those communities near water bodies, migration and land acquisition from safer areas and even in other districts was common practice. For example, people from Pallisa reported moving to nearby districts like Serere. Although they still said this was ineffective, use of crop rotation since the soils had lost fertility. Planting quick maturing and drought-resistant crops, like the peanut variety "Serenut," was reported in Kumi and Serere, and eating once a day during a food shortage, or the use of relief foods from the government for seriously affected communities. Some people often resort to offering cheap labor to those who can provide for their needs, as was also reported by Hisali et al. (2011). Others have reduced the number of domestic animals, especially cattle, goats, and sheep, due to shortage of grazing land, especially in Serere, Kumi, Ngora, and Soroti, which used to be semi-nomadic. Others have resorted to dairy farming, reared on the zero-grazing regime, reducing the number of animals. People hire gardens for a season(s) from those with more land, while others practice aquaculture in wetlands as an alternative livelihood. Small scale irrigation, using valley tanks and solar-powered pumps like in Limoto wetland in Pallisa, has been used to mitigate LULC change and CC impacts. The respondents, however, wanted more significant irrigation projects from the MWE. In Kameke Pallisa, the Kameke wetland was protected by the local community, as they appreciated its value, a rare positive occurrence that needs to be promoted in other areas. Other farmers have formed cooperative societies like Pallisa to improve their income through agriculture. Unfortunately, some families give up their girl children to early marriages to earn money to live on, and participants also reported child labor. Given the increasing community and individual conflicts due to the shortage of land resources, the communities adopted several conflict resolutions approaches, including mediation. It was important to note that the elite need consultancies to develop these measures, but the local communities apply them regularly. They may not have the resources to implement all their coping mechanisms, but they have a wealth of experience that needs to be tapped for better LULC change and CC management.

The local communities expressed challenges about coping mechanisms; these included the unreliability of fast-maturing, drought-resistant crops and exotic tree varieties. Participants in Kumi, Serere, and Pallisa districts noted that the improved crops (cassava and groundnuts) showed a reduction in productivity within two years and were vulnerable to pests and diseases. Meanwhile, exotic trees were destructive to the environment. Relief food for the hungry was not always forthcoming unless major disasters, like landslides in Bududa, attracted the national public concerns and humanitarian response from the central government. Some interventions, like irrigation at a small scale, needs capital, which was not readily available for the local farmers unless external assistance was offered.

Participants agreed that family planning was a better approach to managing the ever-growing population, but the negative perceptions prevented its implementation. Negative perceptions included adverse effects, including over bleeding, failure to conceive following their use, or giving birth to deformed children, and the demand by men for more children. Relatedly, many women feared that when they adopted family planning measures, their husbands would engage in extra-marital affairs with other women to produce children. There is a need for the Ministry of Health (MoH) to carry out more sensitization to both women and men to assure the public about the benefits and how to mitigate any side effects of different family planning measures. This could help reduce the ever-growing population in the study area, expected to reach over 12 million in 2030 (UBoS 2020). This study has shown that family planning approaches are not being applied.

#### Institutional responses

There were several institutional response activities in the study area led by the MWE, through its regional offices under KWMZ. This office works with other agencies like NEMA, NFA; ministries like Ministry of Trade and Cooperatives (MTC); Local governments (LGs); research institutions like National Semi-Arid Resources Research Institute (NaSARRI) in Serere, universities, religious and cultural institutions, local councils (LCs) and the media. These institutions work together with various projects which are focused on mitigation of LULC changes and CC impacts. The sources of funds are mainly from the Government of Uganda (GoU), grants, and donor funds acquired through projects, like the Enhancing Resilience of Communities to Climate Change through Catchment Based Integrated Management of Water and Related Resources in Uganda (EURECCCA), funded by Green Adaptation. Others were Green Future Farming, AID environment, funded by the Dutch government, Integrated Water Management Development Project (IWMDP), funded by World Bank, International Union for conservation of nature (IUNC), funded by IUCN, and Support to resilient initiatives in vulnerable entities (STRIVE) project, funded by Water Aid. Kyoga Water Management Zone was also a government decongested agency in the east, receiving funds from the government and other development partners. These projects are operating in Awoja and Mpologoma catchments in areas of agriculture, CC resilience, drawing catchment management plans, and water supply.

The responses focused on restoring degraded wetlands, forests, and land, although they had different focus components. Their scope was small, and most of them were pilot projects covering small areas. The MWE, through KWMZ, used funds from the EURECCCA project, acquired from Adaptation Fund through Sahara and Sahel Observatory (OSS), to demarcate and restore some wetlands in Awoja catchment, including Oongino in Kumi, Kamurojo in Serere, Adoka in Ngora, Asubakiakiteng in Katakwi, and Limoto in Pallisa-Kibuku. Sections of riverbanks have also been restored, such as rivers Manafwa, Kere, Tabagon-Chepiakamit, and Siti-Greek, to reduce siltation on the downstream lakes, protect river banks, and reduce floods. Water soil conservation activities by the construction of soil and water retention pits, and flood control structures, were implemented to increase groundwater recharge and soil moisture, increasing crop production. This was aimed at controlling rampant soil erosion, especially on the slopes of Mt. Elgon. Demonstration wetlands were also established in Amurojo, Serere, and Adoka to teach local communities how to co-exist and use wetland resources sustainably. Further, the wetlands department of the MWE has developed an inventory of wetlands with risks in the country and is implementing a global CC project which encourages vegetation growth and tree planting in river banks, wetlands, and bare land.

Communities that depended on these wetlands and riverbanks as their source of livelihoods, hence degraded them, were given alternative livelihoods. These included forming a water and environment cooperatives society revolving funds planned by the EURECCCA project, facilitated by MTC and LGs. Construction of fish ponds in Limoto wetland and introduction of piggery as a source of income was done. Others included mushroom farming in Kumi, Ngora, Soroti, and Serere districts. Restoration and conservation of forests have been done by planting indigenous trees and bamboos on the steep slopes and critically degraded catchments by the EURECCCA project. Individuals, groups, and schools participated, with over 725,000 trees planted and about 660 ha of bare land restored. Seedlings and extension services were provided locally by NaSARRI. Relatedly, the production of energy-saving stoves, called 'Lorena' fire-shielded (target was 4200) to local community women groups was also done to conserve forests and trees, improve health by reducing smoke and save time used while looking for firewood. Most interventions have not emphasized measures that encourage terracing in the steep slopes, yet they are essential.

Response contributions of other projects like IUCN in developing catchment management plans (CMPs) for implementation by other projects were necessary. Projects like IWMDP, and STRIVE focused on increasing community resilience to CC by supplying drinking water to households and livestock to mitigate water scarcity caused by CC impacts. These interventions were vital in mitigating the impacts of LULC change and CC and achieving sustainable development goals (SDGs). Goal 6, focused on clean water and sanitation for all in 2030, and goal 15, aimed at protecting, restoring, and promoting sustainable use of terrestrial ecosystems, especially forests and land, including combating desertification and reversing biodiversity loss (UNCCP 2018), are prime targets. However, during the FGDs and KIIs, communities appreciated the Lorena energy-saving stoves and recognized tree planting efforts, although they said the survival rate was low.

#### Policy and regulation response

Uganda has good Policies, Acts of Parliament, and Regulations regarding environment protection and CC; the challenge is their weak implementation. If they were implemented effectively, the observed adverse impacts would be reduced. For example, the Uganda National Policy on Climate Change calls on all stakeholders to manage CC impacts and causes by promoting a green economy for sustainable development (UNCCP 2018), which is not being realized. Uganda is also a signatory to many CC conventions and protocols, like the Kyoto protocol of 1997, on the reduction of greenhouse gas emissions and the Rio Summit of 1992. The National Environment Act, Cap 153, through the National Environment (wetlands, riverbanks, and lakeshores management) regulations, No. 3 of 2000 sets buffer zones for rivers, and wetlands at 100 m, lakes at 200 m (NER 2000), but these are not being followed. The National Forestry and Tree Planting Act of 2003 encourages all people to plant trees and also prohibits the destruction of forests, except under authorization by the responsible authorities. National Agricultural Policy recognizes agriculture as a driver of national economic growth, the most significant source of food and employment. It also recognizes the vital role environment and natural resources play in achieving agricultural objectives. It, therefore, recommends sensitization of the population on the use and conservation of the critical resources, which are soils and water, through extension services (NAP 2013). The National Urban Policy (NUP), through the Town and Country Act of 2000, the National Urban Policy (NUP), and the Physical Planning Act of 2010, are responsible for urban land use and planning (NUP 2017). The policy seeks to promote urban environmental protection and conservation and develop adaptation and mitigation measures against CC. Through the National Environment (standards for discharge for effluents into water or land) Regulations (NER 2020), the Water Act prohibits the discharge of industrial and domestic point source effluents into the environment or water that does not meet the set standards. But most of the effluent quality of the domestic wastes in the study area did not meet these standards, over the years, without any punitive action taken against the companies responsible.

The most significant challenge is the implementation of these legislations since the enforcement arms are either missing or weak. Where the penalties are provided for defaulters, they are not adequately punitive or are not enforced. Meanwhile, some of the provisions of legislation need to be revised to include critical missing areas. For example, the national forestry and tree planting Act should include a section on the follow-up of the people or organizations that plant trees to make sure trees are cared for and survive. Local communities also recommended punishment for individuals and organizations that fail to care for trees and reward the responsible tree planters. There should be regulations on the application of agrochemicals to operationalize National Agricultural policy since there are fake products in the market. This study has shown that Uganda has well-established top-down mechanisms for dealing with LULC change and CC impacts, but are not strictly implemented. This is in contrast to countries like China, which operates mostly a top-down approach but with strict enforcement (Liu et al. 2018). The bottom-up approach is weak in Uganda, a gap that could be due to a shortage of knowledge, and scientific information that this research is trying to provide. There is a need to strengthen both bottom-up and top-down approaches and align them to work together to achieve long-lasting sustainable management and mitigation of LULC change and CC impacts, as pointed out by Reed et al. (2006), Papageorgiou and Kontogianni (2012) and Gray et al. (2014). The focus should be to allow science to speak to policy, to bridge the gap that currently exists between the two paradigms, a finding supported by other studies (Ness et al. 2010; Papageorgiou and Kontogianni 2012; Wantzen et al. 2019).

#### Challenges of mitigation of land use, land cover, and climate change impacts

The institutional response interventions were short-lived and only covered small areas as pilot projects. Therefore, there is a need to expand them, which requires more funds given the expansive nature of LULC change and CC impacts. When the interventional projects reach their lifetime, it is often expected that GoU, through MWE, LGs, and communities, would continue implementing the objectives to ensure the sustainability of their outputs, but this is seldom the case due to a shortage of technical, financial, and infrastructural resources. As a result, in most cases, the end of pilot projects lifespan implies the end of implementing their respective ideas, hence limiting their impact. Meanwhile, the Covid-19 pandemic was one of the challenges that affected the effective implementation and supervision of the project activities. Shortage of land, lack of alternative livelihoods, and the ever-growing population have made the population encroach on protected areas. Hence, the restoration of wetlands and forests is not entirely accepted by local communities, even when involved in these interventions. Local communities often have resistance and violent confrontation against law enforcement officers and intervention staff, exposing them to danger during demarcation exercises, as reported in Adoka and Oongino villages during wetland demarcation and restoration.

Since the constitution gives land rights to individuals, the public often sees restoration interventions as schemes to 'grab' land and are resolutely resisted. Climate change itself was identified as a challenge to the interventions because prolonged dry weather affects the survival of planted trees. Also, superstitious beliefs and attitudes of some of the members of the communities, which are generally "resigned to fate as it were" are unhelpful for conservation efforts since they do not accept that interventions such as tree planting and restoration of wetlands can mitigate against effects of LULC change and CC impacts. Therefore, to ensure the adoption of recommended interventions by local communities, such attitudes need to be changed through sensitization, which takes time. In some cases, seedlings were supplied to the communities during the dry season or towards the end of rainy seasons, which would substantially reduce the chance of survival since there were no options for irrigation. New fast-growing crops promoted as coping measures against CC were prone to pests and diseases, thus requiring the use of agrochemicals to ensure good production. Therefore, the communities recommended indigenous trees, such as fruit trees, and reintroducing indigenous crop varieties, like cassava, including cash crops, like cotton.

There was a strong sentiment by institutions and the local communities on political interference of environmental management activities. Politicians often conveyed conflicting ideas and signals to the public to gain popularity, leading to incitement, resistance to restoration, and conservation efforts by the government and other stakeholders. These political leaders often hold more power over their constituents and are respected by the communities, who are therefore negatively influenced, leading to low adoption of appropriate LULC and CC mitigation measures. This was reported in Ngora, Pallisa, Kibuku, and Kumi districts.

## Conclusion

The LULC changes and CC have been proven by both scientific and local community perceptions in the Lake Kyoga basin. Whereas population was the main underlying driver of LULC change and CC, agriculture was the primary pressure on the natural resources. For management purposes, the study of LULC change and CC together, especially at the local level, should be the new paradigm shift in identifying their drivers and impacts on water and the environment. It is also vital to integrate pure scientific approaches and social sciences when coming up with mitigation measures against impacts of LULC changes and CC. This is critical since scientific study alone without integrating the social knowledge is inadequate in developing local, sustainable management tools. LULC change alone would have limited impacts, especially in water without CC. CC exacerbates impacts of the degradation of land by helping to deliver nutrients and sediments into the receiving water bodies through rivers and streams. There any efforts to mitigate LULC change impacts should incorporate CC. There were many impacts of LULC change and CC in the study area that were articulated by the local communities. These include displacements, deaths, hunger, poverty, damage to infrastructure, loss of biodiversity, and conflicts. Although policies and legislation that mitigate drivers and impacts of LULC change and CC are available, they are not being applied fully. The use of cultural values to manage and protect natural resources as brought forth by this study is one of the management measures that should be pursued. The local communities are rich in knowledge about environmental and CC threats. A lack of resources and leadership only challenges them to develop sustainable interventions and mitigation measures.

FGDs and KIIs are key social science approaches that should become key in identifying and developing bottom-up sustainable management approaches as they allow interaction with the affected local communities, thus integrating their valuable perceptions on any interventions from top-down scientific expert and policy lead measures. This study has underscored the need for an integrated approach to managing natural resources, as the threats do not work in isolation but rather synergize to cause significant impacts. The Ministry of Health needs to sensitize the public on the advantages of family planning since the population is the primary cause of LULC change.

## Recommendations for management options

Most of the response projects have proved successful; however, they had a short lifespan and covered small areas as pilots. For example, the soil water conservation practice done by the construction of soil and water retention reservoirs and flood control structures was at the demonstration pilot level. This should be expanded to cover hilly and steep slope areas of Bukwo, Bududa, Kween, Kapchorwa, and Sironko districts. Demarcation and restoration of wetlands and forests areas should be done using available regulations, and local communities should be encouraged to plant trees in woodlots. Terracing on the steep slopes of Mt. Elgon should be encouraged and done to reduce soil erosion. Therefore, MWE needs to design more significant conservation projects in collaboration with other line ministries like MAAIF, Ministry of Finance Planning and Economic Development (MFPED), and lobby funds locally and internationally for their implementation. This will mitigate against pollution of rivers, wetlands, and Lake Kyoga.

Before any restoration of wetlands, forests, and degraded lands is undertaken, it is essential to seek community buy-in, support, and participation. The leaders (political, religious, and cultural) should be consulted and coopted. Sound and viable alternative sources of income need to be put in place and agreed upon by the affected communities. If necessary, bylaws may be drafted to punish members, including leaders who deviate from abiding by agreed steps for respective projects.

Integration of cultural values into the management of natural resources should be given priority. Most communities respect cultural values; for example, in Pallisa, some hills were protected as cultural celebrations and sacrifice areas. In Nakapiripirit, cutting big trees was prohibited as they were revered as having "gold" and attracting "rains,"; while in Bududa, wetlands were treated as cultural grounds. When these cultural beliefs are integrated into natural resources management, it will be a better local solution; thus, cultural leaders are critical in mitigation measures.

Since food production is the most considerable pressure of LULC change and CC, expansion of irrigation should be prioritized by the MWE. Climate change in the form of droughts and heavy rains affects the study area; rainwater storage during excess rains through the construction of water reservoirs, which would be used for irrigation, domestic and animal watering during droughts should be done. Communities also recommended the growing of upland rice to reduce pressure on wetlands. Further, the local communities recommended that irrigation programs be ensured for both individuals and groups, not the current MWE policy of prioritizing groups only.

There should be reviews of policies, laws, and regulations to match the current LULC changes and CC impacts in the study area and throughout the country since most of these policies were made long ago. The enforcement sections should be included in the regulations and budgeted for if the legislation results are to be realized on the ground. Any policy reviews, legislation, and regulations should take care of the bottom-up paradigm for sustainable management solutions. For example, the National Forestry and Tree planting Act should include in its regulation sections that force people to plant trees, like the principle of "cut one tree, plant two" Planting trees in land boundaries, at roadsides in towns, health centers, hospitals, schools, and peoples' compounds should be regulated. Recognition of the compliant individuals and institutions and punishment for defaulters should be part of the regulation. Therefore, the integrated approach is key to achieving holistic and sustainable management of LULC changes and CC. The Ministry of health should be instrumental in promoting family planning in the communities. There should be some incentives for families that adopt family planning.

Search for markets and value addition to agricultural products should be prioritized to get the farmers out of poverty, which was identified as one of the pressures for LULC changes and CC. This could be achieved by reintroducing cooperative societies, a move the government seems to pursue as recommended by the National Agricultural Policy. There should be regulation by MAAIF on the use of agrochemicals that are not being regulated. Otherwise, with the overall poverty levels in the country, natural resources will continue to degrade.

The communities also requested for recruitment of more agricultural extension workers by MAAIF and their deployment at the sub-county level. Key informants had fond memories of over 40 years ago when extension workers worked closely with farmers and were helpful. They said the current extension workers are not in touch with the farmers and are thin on the ground.

Other recommendations from the study included zoning of the country agriculturally according to the suitable crops/animals for the particular climate and soils. Artisanal mineral mining activities, especially in the Karamoja and Tororo areas, are not conventional; communities complained of pollution from the crude mining methods. Mining should be formalized, and conventional approaches should be applied to reduce pollution of the environment and water resources.

The MWE, through Uganda National Meteorological Authority (UNMA), should improve the accuracy of weather forecasts and broaden the delivery of the forecasts to help farmers plan appropriately. The use of print media, television, radio, and social applications to pass out information to the communities should be encouraged. This study is unique to this area of study, and its findings are relevant since they are from the local communities; it should therefore be replicated in other parts of the country and region. Policy-makers and analysts, water, environment.

## Data Availability

Part of the data used in this research is available with the corresponding author on request. The Lake Kyoga water level data can be obtained from the Ministry of Water and Environment (MWE) on request. The Ministry can be contacted on the website https://www.unma.go.ug/. P.O. Box 20026, Kampala, Uganda.

## References

[CR1] Agyemang I (2012). Assessing the driving forces of environmental degradation in Northern Ghana: community truthing approach. Afr J Hist Cult.

[CR2] Agyemang, I., McDonald, A., & Carver, S. (2007). Application of the DPSIR framework to environmental degradation assessment in northern Ghana. In: Natural Resources Forum (Vol. 31, No. 3, pp. 212–225). Oxford: Blackwell Publishing Ltd.

[CR3] Akhtar A, Rashid SM, Bhat MS, Sheikh AH (2011). Impact of land use/land cover dynamics on Himalayan wetland ecosystem. J Exp Sci.

[CR4] Alemayehu F, Taha N, Nyssen J, Girma A, Zenebe A, Behailu M, Poesen J (2009). The impacts of watershed management on land use and land cover dynamics in Eastern Tigray (Ethiopia). Resour Conserv Recycl.

[CR5] Amjath-Babu TS, Krupnik TJ, Aravindakshan S, Arshad M, Kaechele H (2016). Climate change and indicators of probable shifts in the consumption portfolios of dryland farmers in Sub-Saharan Africa: implications for policy. Ecol Ind.

[CR6] Anaba LA, Banadda N, Kiggundu N, Wanyama J, Engel B, Moriasi D (2016). Application of SWAT to assess the effects of land-use change in the Murchison Bay catchment in Uganda. CWEEE.

[CR7] Andama M, Ongom R, Lukubye B (2017). Proliferation of *Salvinia molesta*at Lake Kyoga landing sites as a result of anthropogenic influences. J Geosci Environ Prot.

[CR8] Ashfaq MY, Al-Ghouti MA, Qiblawey H, Zouari N, Rodrigues DF, Hu Y (2019). Use of DPSIR framework to analyze water resources in Qatar and overview of reverse osmosis as an environment-friendly technology. Environ Prog Sustainable Energy.

[CR9] Belay KT, Van Rompaey A, Poesen J, Van Bruyssel S, Deckers J, Amare K (2015). Spatial analysis of land cover changes in Eastern Tigray (Ethiopia) from 1965 to 2007: are there signs of a forest transition?. Land Degrad Dev.

[CR10] Bell S, Morse S (2001). Breaking through the glass ceiling: who really cares about sustainability indicators?. Local Environ.

[CR11] Bisaro A, Kirk M, Zdruli P, Zimmermann W (2014). Global drivers setting desertification research priorities: insights from a stakeholder consultation forum. Land Degrad Dev.

[CR12] Bone RA, Parks KE, Hudson MD, Tsirinzeni M, Willcock S (2017). Deforestation since independence: a quantitative assessment of four decades of land-cover change in Malawi. Southern for J for Sci.

[CR13] Bremner J, López-Carr D, Suter L, Davis J (2010). Population, poverty, environment, and climate dynamics in the developing world. Interdiscip Environ Rev.

[CR14] Bu H, Meng W, Zhang Y, Wan J (2014). Relationships between land use patterns and water quality in the Taizi River basin, China. Ecol Ind.

[CR15] Camberlin, P., (2009). Nile Basin Climates. In: The Nile: origin, environments, limnology and human use. In: Henri DJ (ed) Vol. 89. Springer Science & Business Media, Berlin, Germany. pp 307–333

[CR16] Crecious H, Lazarus C (2013). Human perceptions on degradation of wetland ecosystems: the case of Magwenzi Wetland in Chivi District; Zimbabwe. GJGES.

[CR17] De Sherbinin A, VanWey LK, McSweeney K, Aggarwal R, Barbieri A, Henry S, Hunter LM, Twine R (2008). Rural household demographics, livelihoods, and the environment. Glob Environ Chang.

[CR18] Donohue I, McGarrigle ML, Mills P (2006). Linking catchment characteristics and water chemistry with the ecological status of Irish rivers. Water Res.

[CR19] Dregne HE (2002). Land degradation in the drylands. Arid Land Res Manag.

[CR20] Dzoga M, Simatele DM, Munga C, Yonge S (2020). Application of the DPSIR framework to coastal and marine fisheries management in Kenya. Ocean Sci J.

[CR21] Elliott M (2002). The role of the DPSIR approach and conceptual models in marineenvironmental management: an example for offshore wind power. Marine pollution bulletin.

[CR22] Farauta BK, Egbule CL, Agwu AE, Idrisa YL, Onyekuru NA (2012). 'Farmers' adaptation initiatives to the impact of climate change on agriculture in northern Nigeria. J Agric Ext.

[CR23] Fatumah N, Tilahun SA, Mohammed S (2020). Effect of tillage systems and tillage direction on soil hydrological properties and soil suspended particle concentration in arable land in Uganda. Heliyon.

[CR24] Francisco HA (2008). Adaptation to climate change: needs and opportunities in Southeast Asia. ASEAN Econ Bull.

[CR25] Gabrielsen P, Bosch P (2003). Environmental Indicators: Typology and Use in Reporting.

[CR26] Gebremedhin S, Getahun A, Anteneh W, Bruneel S, Goethals P (2018). A drivers-pressure-state-impact-responses framework to support the sustainability of fish and fisheries in Lake Tana. Ethiopia Sustainability.

[CR27] Geist HJ, Lambin EF (2002). Proximate Causes and Underlying Driving Forces of Tropical DeforestationTropical forests are disappearing as the result of many pressures, both local and regional, acting in various combinations in different geographical locations. Bioscience.

[CR28] Gessesew WS (2017). Application of DPSIR framework for assessment of land degradation: a review. Forest.

[CR29] Glaser BG, Strauss AL, Strutzel E (1968). The discovery of grounded theory; strategies for qualitative research. Nurs Res.

[CR30] Gondwe MF, Cho MA, Chirwa PW, Geldenhuys CJ (2019). Land use land cover change and the comparative impact of co-management and government-management on the forest cover in Malawi (1999–2018). J of Land Use Science.

[CR31] Goudswaard PC, Katunzi EFB, Wanink JH, Witte F (2011). Distribution of Nile perch Lates niloticus in southern Lake Victoria is determined by the depth and dissolved oxygen concentrations. Afr J Aquat Sci.

[CR32] Gray SRJ, Gagnon AS, Gray SA, O'Dwyer B, O'Mahony C, Muir D, Gault J (2014). Are coastal managers detecting the problem? Assessing stakeholder perception of climate vulnerability using Fuzzy Cognitive Mapping. Ocean Coast Manag.

[CR33] Grêt-Regamey A, Celio E, Klein TM, Hayek UW (2013). Understanding ecosystem services trade-offs with interactive procedural modeling for sustainable urban planning. Landsc Urban Plan.

[CR34] Hägerstrand T, Buttimer A (2001). A look at the political geography of environmental management. Sustainable landscapes and lifeways: scale and appropriateness.

[CR35] Harte J (2007). Human population as a dynamic factor in environmental degradation. Popul Environ.

[CR36] Hisali E, Birungi P, Buyinza F (2011). Adaptation to climate change in Uganda: evidence from microdata. Glob Environ Chang.

[CR37] Hou Y, Zhou S, Burkhard B, Müller F (2014). Socioeconomic influences on biodiversity, ecosystem services, andhuman well-being: A quantitative application of the DPSIR model in Jiangsu, China. Science of the TotalEnvironment.

[CR38] Huong HTL, Pathirana A (2013). Urbanization and climate change impacts on future urban flooding in Can Tho city, Vietnam. Hydrol Earth Syst Sci.

[CR39] IPPC (2007). Summary for Policy makers-level. In: Climate change: Impacts, Adaptation, and Vulnerability. Contribution of Working Group II to the Fourth Assessment Report of the Intergovernmental Panel on Climate. Cambridge University Press, UK.

[CR40] Juhé-Beaulaton D.2006.Economic and social issues around sacred woods and the "conservation of biodiversity", Benin, Burkina Faso, and Togo. Proceedings of the IFB workshop, 2006, Dynamics of biodiversity and modalities of access to environments and resources, Fréjus 79 September 2005, Paris, IFB:6872.

[CR41] Kabubo-Mariara J (2007). Land conservation and tenure security in Kenya: Boserup's hypothesis revisited. Ecol Econ.

[CR42] Kelble, C. R., Loomis, D. K., Lovelace, S., Nuttle, W. K., Ortner, P. B., Fletcher, P., ... & Boyer, J. N. (2013). The EBM-DPSER conceptual model: integrating ecosystem services into the DPSIR framework. PloS one, *8*(8), e70766. 10.1371/journal.pone.0070766 PMC3741316 23951002

[CR43] Keskinen M, Chinvanno S, Kummu M, Nuorteva P, Snidvongs A, Varis O, Västilä K (2010). Climate change and water resources in the Lower Mekong River Basin: putting adaptation into the context. J Water Climate Change.

[CR44] Kirui, O., & Mirzabaev, A. (2015). Drivers of land degradation and adoption of multiple sustainable land management practices in Eastern Africa (No. 1008–2016–80052).

[CR45] Kokou K, Sokpon N (2006). The sacred forests of the Dahomey corridor. Woods Forests Tropics.

[CR46] Kosamu IBM, Makwinja R, Kaonga CC, Mengistou S, Kaunda E, Alamirew T, Njaya F (2022). Application of DPSIR and Tobit Models in Assessing FreshwaterEcosystems: The Case of Lake Malombe. Malawi. Water.

[CR47] Kristensen, P. (2004). The DPSIR Framework, workshop on a comprehensive/detailed assessment of the vulnerability of water resources to environmental change in Africa using river basin approach. UNEP Headquarters, Nairobi, Kenya.

[CR48] Kundzewicz ZW, Kanae S, Seneviratne SI, Handmer J, Nicholls N, Peduzzi P, Mechler R, Laurens MB, Arnell N, Mach K, Muir-Wood R, Brakenridge GR, Kron W, Benito G, Honda Y, Takahashi K, Sherstyukov B (2014). Flood risk and climate change: global and regional perspectives. Hydrol Sci J.

[CR49] Lal, A., (2016). Salvinia molesta : an assessment of the effects and methods of eradication. Master' 'Master's Proj. Capstones 572.

[CR50] Lambin EF, Geist HJ (2008). Land-use and land-cover change: local processes and global impacts.

[CR51] Lambin EF, Turner BL, Geist HJ, Agbola SB, Angelsen A, Bruce JW, Coomes OT, Dirzo R, Fischer G, Folke C, George PS, Homewood K, Imbernon J, Leemans R, Li XB, Moran EF, Mortimore M, Ramakrishnan PS, Richards JF, Skanes H, Steffen W, Stone GD, Svedin U, Veldkamp TA, Vogel C, Xu J (2001). The causes of land-use and land-cover change: moving beyond the myths. Glob Environ Chang.

[CR52] Levy PS, Lemeshow S (2013). Sampling of populations: methods and applications.

[CR53] Liu X, Liu H, Chen J, Liu T, Deng Z (2018). Evaluating the sustainability of marine industrial parks based on the DPSIR framework. J Clean Prod.

[CR54] Madriz E (2000) Focus Groups in Feminist Research. In: Denzin NK and Lincoln YS (eds) Handbook of Qualitative Research 2nd edition. pp 835–850

[CR55] Makwinja R, Kaunda E, Mengistou S, Alamirew T (2021). Impact of land use/land cover dynamics on ecosystem service value—a case from Lake Malombe, Southern Malawi. Environ Monitor Assess.

[CR56] Martins JH, Camanho AS, Gaspar MB (2012). A review of the application of driving forces–Pressure–State–Impact–Response framework to fisheries management. Ocean Coast Manag.

[CR57] Matagi SV (2002). Some issues of environmental concern in Kampala, the capital city of Uganda. Environ Monit Assess.

[CR58] Maxwell JA (2012). Qualitative research design: an interactive approach.

[CR59] Mbogga, M. S. (2012). Climate profiles and climate change vulnerability assessment for the Mbale region of Uganda. UNDP Consultancy report. Kampala, Uganda.

[CR60] McFarland DG, Nelson LS, Grodowitz MJ, Smart RM, Owens CS (2004). Salvinia molesta DS Mitchell (giant Salvinia) in the United States: A review of species ecology and approaches to.

[CR61] McKinney ML (2002). Urbanization, Biodiversity, and Conservation. The impacts of urbanization on native species are poorly studied, but educating a highly urbanized human population about these impacts can greatly improve species conservation in all ecosystems. Bioscience.

[CR62] Ministry of Agriculture, Animal Industry and Fisheries (MAAIF) (2020) Statement on the Fish Kills in Lakes Victoria and Kyoga, Kampala, Uganda. https://www.nema.go.ug. Accessed 10 Oct 2021

[CR63] Ministry of Water and Environment (MWE). Integrated territorial climate plan 2014–2029 for Mbale region, Uganda (Bududa, Mbale, and Manafwa districts), Kampala, Uganda. 2013.

[CR64] Ministry of Water and Environment (MWE), (2015). Catchment management for Awoja catchment. Kampala Uganda. https://www.mwe.go.ug/. Accessed 26 Aug 2021.

[CR65] Ministry of Water and Environment, 2018a. Mpologoma Catchment Management Plan. Ministry of Water and Environment, Kampala, Uganda.

[CR66] Ministry of Water and Environment (MWE) (2020) Water and Environment Sector Performance Report, Kampala Uganda.https://www.mwe.go.ug/. Accessed 26 Aug 2021

[CR67] Miranda LE, Andrews CS, Kröger R (2014). Connectedness of land use, nutrients, primary production, and fish assemblages in oxbow lakes. Aquat Sci.

[CR68] Mustard JF, Defries RS, Fisher T, Moran E, Gutman G, Janetos AC, Justice CO, Moran EF, Mustard JF, Rindfuss RR, Skole D, Turner BL, Cochran MA (2005). Land use and land cover change pathways and impacts. LandChange Science: Observing, Monitoring, and 16 Understanding Trajectories of Change on the Earth's Surface.

[CR69] Namaalwa S, Funk A, Ajie GS, Kaggwa RC (2013). A characterization of the drivers, pressures, ecosystem functions, and services of Namatala wetland, Uganda. Environ Sci Policy.

[CR70] National Agricultural Policy (NAP). (2013). Ministry of Agriculture, Animal Industry and Fisheries (MAAIF). Kampala, Uganda.

[CR71] National Environment (Wetlands; River Banks and Lake Shores Management) Regulations, (2000). Ministry of Water and Environment (MWE). Kampala, Uganda.

[CR72] National Environment Regulations (NER) (2020) Standards for Discharge of Effluent into Water or Land. Kampala, Uganda

[CR73] The National Urban Policy (NUP) (2017). Transformed and Sustainable Urban Areas’.

[CR74] Ness B, Anderberg S, Olsson L (2010). Structuring problems in sustainability science: the multi-level DPSIR framework. Geoforum.

[CR75] Nkonya, E., Gerber, N., von Braun, J., & De Pinto, A. (2011). Economics of land degradation: the costs of action versus inaction, IFPRI issue brief no. 68.

[CR76] Nyumba OT, Wilson K, Derrick CJ, Mukherjee N (2018). The use of focus group discussion methodology: Insights from two decades of application in conservation. Methods Ecol Evol.

[CR77] Obubu JP, Mengistou S, Fetahi T, Alamirew T, Odong R, Ekwacu S (2021). Recent climate change in the Lake Kyoga Basin, Uganda: an analysis using short-term and long-term data with standardized precipitation and anomaly indexes. Climate.

[CR78] Obubu JP, Mengistou S, Odong R, Fetahi T, Alamirew T (2021). Determination of the connectedness of land use, land cover change to water quality status of a shallow lake: a case of Lake Kyoga Basin, Uganda. Sustainability.

[CR79] OECD. Environmental indicators: development, measurement, and use; Reference Paper; OECD: Paris, France, 2003.

[CR80] Ogutu-Ohwayo R, Odongkara K, Okello W, Mbabazi D, Wandera SB, Ndawula LM, Natugonza V (2013). Variations and changes in habitat, productivity, the composition of aquatic biota and fisheries of *L. Kyoga* system: lessons for management. Afr J Aquat Sci.

[CR81] Ojara MA, Lou Y, Aribo L, Namumbya S, Uddin MJ (2020). Dry spells and probability of rainfall occurrence for Lake Kyoga Basin in Uganda East Africa. Nat Hazards.

[CR82] Oliver JD (1993). A review of the biology of giant *Salvinia*.

[CR83] Onwuegbuzie AJ, Dickinson WB, Leech NL, Zoran AG (2009). A qualitative framework for collecting and analyzing data in focus group research. Int J Qual Methods.

[CR84] Osbahr H, Dorward P, Stern R, Cooper S (2011). Supporting agricultural innovation in Uganda to respond to climate risk: linking climate change and variability with farmer perceptions. Exp Agric.

[CR85] Palmer MA, Hondula KL, Koch BJ (2014). Ecological restoration of streams and rivers: shifting strategies and shifting goals. Annu Rev Ecol Evol Syst.

[CR86] Papageorgiou, E., & Kontogianni, A. (2012). Using fuzzy cognitive mapping in environmental decision making and management: a methodological primer and an application. International perspectives on global environmental change, 427–450. 10.5772/29375

[CR87] Patton MQ (1990). Qualitative evaluation and research methods.

[CR88] Pingali, P., Schneider, K., & Zurek, M. (2014). Poverty, agriculture and the environment: The case of Sub-Saharan Africa. Marginality: Addressing the nexus of poverty, exclusion, and ecology, Springer, Netherlands, 151-68. 10.1007/978-94-007-7061-4_10

[CR89] Pinto R, De Jonge VN, Neto JM, Domingos T, Marques JC, Patrício J (2013). Towards a DPSIR-driven integration of ecological value, water uses, and ecosystem services for estuarinesystems. Ocean & Coastal Management.

[CR90] Porta J, Claret RMP (2011). DPSIR analysis of land and soil degradation in response to changes in land use. SJSS.

[CR91] Rawat JS, Kumar M (2015). Monitoring land use/cover change using remote sensing and GIS techniques: A case study of Hawalbagh block, district Almora, Uttarakhand, India. The Egyptian Journal of Remote Sensing and Space Science.

[CR92] Reed MS, Fraser ED, Dougill AJ (2006). An adaptive learning process for developing and applying sustainability indicators with local communities. Ecol Econ.

[CR93] Reynolds JF, Smith DMS, Lambin EF, Turner BL, Mortimore M, Batterbury SP, Walker B (2007). Global desertification: building a science for dryland development. Science.

[CR94] Ricaurte LF, Wantzen KM, Agudelo E, Betancourt B, Jokela J (2014). Participatory rural appraisal of ecosystem services of wetlands in the Amazonian Piedmont of Colombia: elements for a sustainable management concept. Wetlands Ecol Manage.

[CR95] Sandelowski M, Given LM (2008). Theoretical saturation. The Sage encyclopedia of qualitative methods.

[CR96] Sekovski I, Newton A, Dennison WC (2012). Megacities in the coastal zone: Using a driver-pressure-state-impact-response framework to address complex environmental problems. Estuar Coast Shelf Sci.

[CR97] Solehana L, Asrori A, Usman A (2019). The development of e-learning teaching material based on edmodo on basic competencies of national integration at class X of senior high school. JETL.

[CR98] Sun R, Chen L, Chen W, Ji Y (2013). Effect of land-use patterns on total nitrogen concentration in the upstream regions of the Haihe River Basin China. Environ Manag.

[CR99] Sun S, Wang Y, Liu J, Cai H, Wu P, Geng Q, Xu L (2016). Sustainability assessment of regional water resources under the DPSIR framework. J Hydrol.

[CR100] Tang Z, Engel BA, Pijanowski BC, Lim KJ (2005). Forecasting land-use change and its environmental impact at a watershed scale. J Environ Manag.

[CR101] Tien NN, Thuy NTT (2020). Impact of FDI on economic growth from the sustainable development perspective: a case study from the assessment in the middle of Vietnam. Kasetsart J Soc Sci.

[CR102] Timmerman JG, Beinat E, Termeer CJAM, Cofino WP (2011). Developingtransboundary river basin monitoring programs using the DPSIR indicator framework. Journal of Environmental Monitoring.

[CR103] Uganda Bureau of Statistics (UBoS). Uganda Population Projections. Kampala Uganda. 2020. https://www.ubos.org. Accessed 23 Sept 2021.

[CR104] Uganda National Climate Change Policy (UNCCP). (2018). Ministry of Water and Environment, Republic of Uganda, Transformation through Climate Change Mitigation and Adaptation, Kampala, Uganda.

[CR105] UNICEF (2020). Uganda's multidimensional poverty profile, going beyond monetary poverty. Kampala, Uganda. www.unicef.org/uganda. Accessed 08 Nov 2021

[CR106] UNFCCC (United Nations Framework Convention on Climate Change) (2011) Fact sheet: Climate change science - the status of climate change science today. https://unfccc.int/files/press/backgrounders/application/pdf/press_factsh_science.pdf. Accessed 21 June 2021

[CR107] Urama KC, Ozor N (2010). Impacts of climate change on water resources in Africa: the role of adaptation. Afr Technol Policy Studies Netw.

[CR108] Vu QM, Le QB, Frossard E, Vlek PL (2014). Socio-economic and biophysical determinants of land degradation in Vietnam: an integrated causal analysis at the national level. Land Use Policy.

[CR109] Wan R, Cai S, Li H, Yang G, Li Z, Nie X (2014). Inferring land use and land cover impact on stream water quality using a Bayesian hierarchical modeling approach in the Xitiaoxi River Watershed, China. J Environ Manag.

[CR110] Wantzen KM, Alves CB, Badiane SD, Bala R, Blettler M, Callisto M, Cao Y, Kolb M, Kondolf GM, Leite MF, Macedo DR (2019). Urban stream and wetland restoration in the Global South—a DPSIR analysis. Sustainability.

[CR111] Wanda FM, Balirwa JS, Ogwanga JA, Moro R, Amondito BA (2015). New Water Weed, Giant Salvinia (Salvinia molesta), Invades Lake Kyoga, Jinja.

